# Priority Setting for Universal Health Coverage: We Need Evidence-Informed Deliberative Processes, Not Just More Evidence on Cost-Effectiveness

**DOI:** 10.15171/ijhpm.2016.83

**Published:** 2016-06-22

**Authors:** Rob Baltussen, Maarten P. Jansen, Evelinn Mikkelsen, Noor Tromp, Jan Hontelez, Leon Bijlmakers, Gert Jan Van der Wilt

**Affiliations:** ^1^Radboud Institute for Health Sciences, Radboud University Medical Center, Nijmegen, The Netherlands.; ^2^Erasmus MC, University Medical Center Rotterdam, Rotterdam, The Netherlands.; ^3^Harvard T. H. Chan School of Public Health, Harvard University, Boston, MA, USA.; ^4^Africa Centre for Population Health, Mtubatuba, South Africa.

**Keywords:** Universal Health Coverage (UHC), Priority Setting, Cost-Effectiveness Analysis, Evidence-Informed Deliberative Processes, Decision-Making, Legitimacy

## Abstract

Priority setting of health interventions is generally considered as a valuable approach to support low- and middle-income countries (LMICs) in their strive for universal health coverage (UHC). However, present initiatives on priority setting are mainly geared towards the development of more cost-effectiveness information, and this evidence does not sufficiently support countries to make optimal choices. The reason is that priority setting is in reality a value-laden political process in which multiple criteria beyond cost-effectiveness are important, and stakeholders often justifiably disagree about the relative importance of these criteria. Here, we propose the use of ‘evidence-informed deliberative processes’ as an approach that does explicitly recognise priority setting as a political process and an intrinsically complex task. In these processes, deliberation between stakeholders is crucial to identify, reflect and learn about the meaning and importance of values, informed by evidence on these values. Such processes then result in the use of a broader range of explicit criteria that can be seen as the product of both international learning (‘core’ criteria, which include eg, cost-effectiveness, priority to the worse off, and financial protection) and learning among local stakeholders (‘contextual’ criteria). We believe that, with these evidence-informed deliberative processes in place, priority setting can provide a more meaningful contribution to achieving UHC.

## Introduction

In January 2016, the Prince Mahidol Award Conference (PMAC) in Thailand brought together more than 900 delegates from 60 different countries, to discuss priority setting of health interventions to achieve universal health coverage (UHC).^1^ The goal of UHC is to ensure that all people obtain the health services they need, without suffering financial hardship when paying for them.^2^ At the conference, the World Health Organization (WHO) and the Disease Control Priority (DCP) project presented their impressive work to expand the evidence base on the cost-effectiveness of interventions, concentrated on low- and middle-income countries (LMICs).^3,4^ The underlying implicit assumption to these analyses is that priority setting should be geared towards maximisation of population health, and that the provision of more evidence on cost-effectiveness will improve decision-making and lead to better health. This rationale also underpins the development of many international disease control guidelines. For example, the most recent WHO guidelines on when to start antiretroviral therapy for HIV are largely based on the expected epidemiological impact and cost-effectiveness.^5^

In this editorial, we argue that the mere provision of cost-effectiveness information does not adequately support countries to make optimal choices. The reason is that priority setting is in reality a value-laden political process, in which multiple criteria beyond cost-effectiveness are important and stakeholders often justifiably disagree about their relative importance. Instead, we propose the use of ‘evidence-informed deliberative processes’ as an approach that does explicitly recognise priority setting as a political process and an intrinsically complex task. In these processes, deliberation between stakeholder is crucial to identify, reflect and learn about the importance of relevant values, informed by evidence on these values.

We first outline the need for evidence-informed deliberative processes, illustrate this with examples from Indonesia, Thailand, and the Netherlands, discuss how to preserve the use of social core values in these processes, and then elaborate on their use to achieve UHC.

## The Need for Evidence-Informed Deliberative Processes

It has been recognised since long that priority setting is in reality a value-based political process which takes place in an environment of social values and diverging interests.^6-10^ It involves “*pluralistic bargaining between different lobbies, modified by shifting political judgements made in the light of changing pressures*”^11^ and is described as “*a complex interaction of various decisions at diverse levels in the organization. There is no self-obvious set of ethical principles or scientific tools to determine what decisions we should take at various levels.”*^12^

If we agree that priority setting is a value-based political process, this then requires a paradigm shift in how research should approach the challenge of priority setting, addressing two important issues. Firstly, society – including relevant stakeholders such as patients, providers, insurers, and citizens – has a wide range of social values to judge decisions. These go beyond only health maximization as reflected in the criterion ‘cost-effectiveness,’ for example caring for the worse off in society or responsibility for own health.^13-18^ We argue that the whole of these values should be considered when setting priorities. Second, stakeholders often disagree about the importance of these values, and may have good reasons to do so when it comes to setting priorities. In the light of this, Daniels and Sabin have proposed the use of fair processes as an alternative approach to priority setting. In their seminal work on ‘accountability for reasonableness’ (A4R), they argue that it is more likely that stakeholders will agree on a fair process to set priorities than on the use of specific social values – and if they do so, stakeholders are then also more likely to confer legitimacy to the decisions that are made through this process.^10^

Following this logic, and in acceptance of these two issues, the central question in priority setting becomes: ‘How can priority setting processes be organised such that stakeholders confer legitimacy to the decisions that will eventually be taken’? Or in other words: ‘so that they accept these decisions as reasonable?’ Daniels and Sabin propose conditions for transparency, relevance, appeal, and enforcement that processes should meet to achieve legitimacy.^10^ The aim of such processes is to develop a mutual basis for decisions among stakeholders, through the identification, interpretation and deliberation on a range of values that they find important, and informed by evidence where possible.

Where Daniels and Sabin speak of ‘fair processes,’ we preferably name these ‘evidence-informed deliberative processes’ to better position the approach in the present public health debate. On the one hand, these processes are based on deliberation between stakeholders to identify, reflect and learn about the meaning and importance of relevant social values. On the other hand, they are based on rational decision-making – through evidence-informed evaluation of the identified values where possible. We speak of evidence-informed rather than evidence-based evaluation as the former concept leaves ample room for clinical experience as well as the constructive judgements of stakeholders such as practitioners and patients who are in constant interaction and dialogue with one another.^19^ We see ‘evidence-informed’ and ‘deliberation’ as the two essential elements to achieve legitimacy in priority setting. To date, the work on fair processes (or how we name it: evidence-informed deliberative processes), has mainly been theoretical, and we see large potential for their practical application to support countries in their strive for UHC.

## Development of Evidence-Informed Deliberative Processes

We distinguish six steps in the use of evidence-informed deliberative processes (Figure), and these are described in detail elsewhere.^20^ The dotted lines reflect that the process is iterative. The way these steps can be applied in a decision-making context to foster the legitimacy of the eventual decisions, depend on the already existing priority setting process. To illustrate this, we provide two examples: (*i*) a case study to support HIV control in Indonesia, where priority setting has historically been implicit and entire processes need to be established; (*ii*) reimbursement decisions in the Netherlands and Thailand where HTA agencies are firmly established, and processes are relatively well-developed. It is obvious that in the former example, there is a relatively large need and large potential to improve the legitimacy of decisions, as compared to the latter example.

**Figure:**
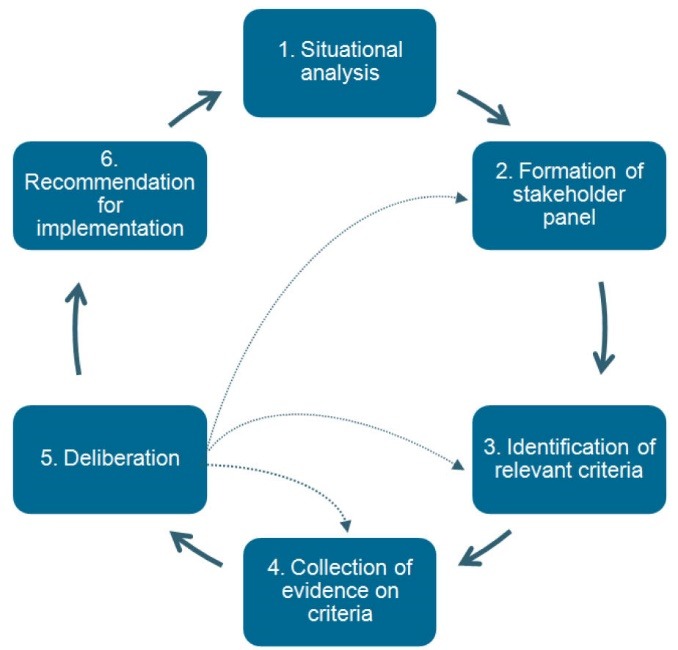
Six Steps of Evidence-Informed Deliberative Processes.

### Supporting HIV Control in Indonesia

We recently supported West Java provincial authorities in Indonesia in the development of their strategic plan on HIV/AIDS.^20-23^ The process included a situational analysis on the current response to the HIV epidemic (step 1); the formation of a multi-stakeholder consultation panel (step 2); the identification of stakeholder values for the most important goals in HIV control, resulting in a set of criteria for priority setting that were considered reasonable by all panel members (step 3); a listing of HIV/AIDS intervention options by the consultation panel, including the collection of evidence to assess their performance (step 4); a deliberative discussion among the consultation panel members on this evidence, in view of their values and interests, to reach agreement on the final rank order of interventions (step 5); and a listing of institutions that would be suitable and/or fund high priority interventions (step 6). The overall aim of the process was to organise priority setting as an interactive learning process, in which the consultation panel refined the participatory steps of identifying, elaborating and deciding on the inclusion of further relevant stakeholders, criteria and evidence. A recent evaluation indicated that panel members were overall positive about the process, as it had improved the quality of decision-making̶especially in terms of use of multiple criteria and concrete evidence, active participation of stakeholders, and transparency of decision-making.^22,23^

Yet, we also recognize that, for logistic and budgetary reasons, it is not feasible to develop such processes from scratch in all decision-making contexts where no priority setting process is in place. Ideally, countries should work towards the development of more generic centrally-led institutionalized processes, which would then be used at decentralised level as guidance for priority setting.^24,25^ We see our work as a stepping stone towards such institutionalized processes, by spelling out important principles, documenting the initial experiences, and thereby creating awareness about its potential and limitations. In this context, we applaud the pioneering work of the International Decision Support Initiative (iDSI), which provides policy-makers at sub-national, national, regional, and international levels with technical support in coordinating priority setting as a means towards achieving UHC.^25^

### Reimbursement Decisions by Health Technology Assessment Agencies

A number of national health technology assessment (HTA) agencies have already important components of evidence-based deliberative processes in place. For example, the Netherlands Health Care Institute (ZINL) employs an appraisal committee that represents the Dutch society and advises the Minister of Health. The committee deliberates on the social value of health technologies on the basis of the available evidence on four generic criteria (effectiveness, cost-effectiveness, necessity, and feasibility), along with other contextual criteria that are considered relevant to the interventions under scrutiny.^26^ In Thailand, the National Health Security Office employs a consultation panel which works with a large group of stakeholders to select interventions for assessment. The panel appraises the interventions on several criteria and deliberates to reach consensus on which interventions should be adopted in the benefit package.^27^ While these agencies employ various elements of evidence-based deliberative processes, they can still improve on other elements eg, stakeholder involvement in the Netherlands. This in order to further foster the legitimacy of their decisions.

From a more methodologically point of view: various approaches to priority setting contain important elements of evidence-informed deliberative processes, including A4R,^10^ multi-criteria decision analysis (MCDA)^28^ and programme budgeting and marginal analysis (PBMA).^29,30^ The frameworks have been applied in various settings.^27,31-36^ The added value of our framework is that it combines these elements in an integrated approach.

## The Use of Social ‘Core’ Values to Achieve Universal Health Coverage

An important issue in the use of evidence-informed deliberative processes is how important social values can be preserved in the process. Stakeholder consultation, especially in the presence of vested interests, does not necessarily capture such public interests.

In its report ‘Making fair choices on the path to UHC,’ the WHO recently proposed the use of ‘cost-effectiveness,’ ‘priority to the worse off,’ and ‘financial protection’ as the three most essential criteria for countries to consider when setting priorities.^18^ We consider these as ‘core’ criteria, representing social values for which there is broad consensus on their importance and which are of generic relevance across countries, disease areas and health interventions. Their identification can be seen as the product of international learning, particularly in academic circles, on priority setting.^37^ One way to preserve these ‘core’ values in evidence-informed deliberative processes is to consider them as mandatory criteria for healthcare priority setting. Another, less stringent option is to use them as ‘opt-out’ criteria, for which a decision-maker should provide compelling arguments when declining them. We argue that the use of evidence-informed deliberative processes would then be instrumental to consider additional criteria for priority setting for which there is no broad consensus or which are only relevant for a particular decision – we call these ‘contextual criteria.’ These can include many criteria, eg, ‘responsibility for own health’ or ‘size of the population affected.’^37^

Above we criticize WHO and the DCP project for its use of cost-effectiveness analysis as the sole criterion. Yet, how we see it, ‘cost-effectiveness’ can very well be a ‘core’ criterion in priority setting but should be interpreted in the context of other stakeholder values. In other words, our critique concerns the dominant use of cost-effectiveness analysis, not cost-effectiveness analysis as such. In fact, the DCP project is now conducting extended cost-effectiveness analysis (ECEA), to also capture the financial protection which interventions offer to target populations.^38^ We applaud this effort as it provides valuable evidence on what we see as one of the core criteria in priority setting to achieve UHC. Yet, we recommend that this DCP work should be coupled with the development of evidence-informed deliberative processes at the country level, to also identify other contextual criteria.^22^

## Our Contribution

As a research group, we carry out various activities under the heading of the REVISE (REthinking the Value of Interventions to improve priority SEtting) project to further develop evidence-informed deliberative processes.^39^ We are:

- developing best practices on the various elements of these processes, eg, on whose values to consider, how to best guide the identification of criteria, and how to deal with vested interests. Our first findings are published in a companion paper;^40^
- collaborating with other disciplines such as public administration and political sciences, to learn from similar processes in other fields;^41,42^
- experimenting with the implementation of processes in various contexts and evaluate these; and
- stimulating the debate between researchers, policy-makers, and society on the need to set legitimate priorities in healthcare.^39^

## Conclusion

It is time to focus on the development of evidence-informed deliberative processes to set legitimate priorities, and we call for more research in this area. We believe that with such processes in place, priority setting can provide an important contribution to achieving UHC.

## Ethical issues

Not applicable.

## Competing interests

Authors declare that they have no competing interests.

## Authors’ contributions

All authors have contributed to the conceptualisation and the writing of this paper.

## Authors’ affiliations

^1^Radboud Institute for Health Sciences, Radboud University Medical Center, Nijmegen, The Netherlands‏. ^2^Erasmus MC, University Medical Center Rotterdam, Rotterdam, The Netherlands. ^3^Harvard T. H. Chan School of Public Health, Harvard University, Boston, MA, USA.‏ ^4^Africa Centre for Population Health, Mtubatuba, South Africa‏.
